# Development and Evaluation of a Nutrition-Centered Lifestyle Medicine Curriculum for Physician Assistant Students

**DOI:** 10.1007/s40670-018-00655-4

**Published:** 2018-12-06

**Authors:** Marianna S. Wetherill, Gracen C. Davis, Krista Kezbers, Valarie Carter, Elizabeth Wells, Mary B. Williams, Shannon D. Ijams, Dominique Monlezun, Timothy Harlan, Lori J. Whelan

**Affiliations:** 1 Hudson College of Public Health, University of Oklahoma - Tulsa Schusterman Center, 4502 E. 41st Street, Tulsa, OK 74135 USA; 2 University of Oklahoma School of Community Medicine, Tulsa, OK 74135 USA; 3 Tulane University School of Medicine, New Orleans, LA 70112 USA

**Keywords:** Physician assistant students, Medical nutrition education, Culinary medicine, Lifestyle medicine

## Abstract

**Background:**

US medical schools are increasingly integrating lifestyle medicine competencies into their academic programs. Yet, physician assistant (PA) academic programs have been slower to respond.

**Methods:**

We developed, implemented, and evaluated a nutrition-centered lifestyle medicine curriculum for 2nd-year PA students (*n* = 24). The 4-week hybrid, 2-credit hour course activities aligned with the American College of Lifestyle Medicine competencies for primary care providers and reinforced four of the Accreditation Standards for PA Education. We combined didactic lectures with weekly hands-on cooking modules from the “Health meets Food” courseware for medical students. We employed a pre-post evaluation design including a comparison group of 2nd-year PA students in a separate program. We assessed changes in personal nutrition behaviors and knowledge and confidence for counseling in nutrition, exercise/physical activity, weight, smoking, and alcohol, using the modified 5A’s framework (assess, advise, agree, assist, and arrange) for lifestyle counseling.

**Results:**

Students receiving the intervention demonstrated significantly higher gains in both knowledge and confidence for the 5A’s of nutrition counseling compared to the control group. Self-reported knowledge and confidence for the 5A’s of counseling for the other lifestyle behaviors similarly improved among the intervention group compared to the control group, but to a lesser extent.

**Conclusion:**

A nutrition-centered lifestyle medicine course can demonstrate PA academic program adherence to accreditation standards, while also introducing students to nutrition and lifestyle medicine competencies. Hands-on experiences that reinforce didactic instruction may maximize student knowledge and self-efficacy for implementing lifestyle medicine into their practice.

## Introduction

Physician assistants (PAs) fill a critical need in the US healthcare system, often serving as the main healthcare provider for patients in a variety of primary and specialty care medical settings. The irrefutable role of nutrition in disease prevention, management, and treatment has resulted in increasing attention by medical school programs to integrate nutrition training into their curricula. More recently, the need to advance the nutrition education in the training of other healthcare professions, including PAs, has been identified [1]. Yet, very few recent studies describe the inclusion of nutrition into PA curricula, with one observational study finding a decline in PA student nutrition-related knowledge, self-perceived proficiency, and attitudes across three cohorts [2].

Nutrition plays a role in all physiologic processes and relates to the prevention and management of nearly every chronic disease state, and thus can be naturally integrated systematically into pre-clinical and clinical PA student coursework. Similar to medical student training programs, nutrition and lifestyle medicine-related curricula can be used to demonstrate adherence to several fundamental accreditation standards for PA programs. Although the term “nutrition” is not explicitly stated in the Accreditation Standards for Physician Assistant Education [3], many of the accreditation competencies required during clinical preparatory instruction implicitly require foundational knowledge and skills in health behavior assessment and patient education. For example, all accredited PA programs are required to provide “instruction in patient evaluation, diagnosis, and management…including acute and longitudinal management with treatment plans [that are] patient centered and inclusive, addressing medical issues, patient education, and referral (B2.05).” Additional competencies that imply nutrition and other lifestyle medicine-related knowledge and skills include the provision of preventive and chronic medical care across the lifespan (B2.06), instruction in the social and behavioral sciences (B2.08), and basic counseling and patient education skills that include behavioral modification to more healthful patterns (B2.09).

*Lifestyle medicine*, as its own discipline, is defined as “the evidence-based practice of helping individuals and families adopt and sustain healthy behaviors that affect health and quality of life.” Examples of target patient behaviors include, but are not limited to, eliminating tobacco use, improving diet, increasing physical activity, and moderating alcohol consumption [4]. The American College of Lifestyle Medicine places a relatively large emphasis on nutrition as compared to other individual health behaviors, and has also provided additional guidance on four core lifestyle medicine competencies for all primary care providers that involve assessment skills, management skills, leadership and role modeling, and the use of office and community support [5]. These competencies are viewed as essential for patient behavior change, since studies suggest that medical providers are more likely to counsel patients on lifestyle behaviors when they perform the behaviors themselves [6], and that patients are more receptive to lifestyle prescriptions when they perceive their providers also perform those behaviors [7] and are confident in the recommendation [8].

The addition of new curriculum requirements may be perceived as a challenge by administrators of PA programs, since PA training involves an intense course load that parallels nearly all competencies included in 4-year medical school degrees, yet averages only 27 months total of pre-clinical and clinical instruction [9]. In a 2000 national study of nutrition education in PA programs, among the 50% of nationally accredited programs that responded, only 29% provided a separate course in nutrition and the methods of teaching nutrition relied heavily on lectures and readings (98%) with infrequent use of hands-on instruction [10]. Many US medical schools have employed culinary medicine, “a new evidence-based field in medicine that blends the art of food and cooking with the science of medicine” to provide students with interactive opportunities for practical learning [11]. A more recent review of nutrition education programs for medical students indicates that effective nutrition education interventions must improve skills, self-efficacy, and attitudes of learners in addition to knowledge [12]. By teaching student learners new skills for preparing healthy and therapeutic foods, culinary medicine teaching approaches may additionally help to achieve these aims.

This paper describes the development and evaluation of a nutrition-centered, lifestyle medicine curriculum for PA students that involves the first PA program in the nation to pilot the “Health meets Food” courseware as a component of its degree requirements. The goals of the curriculum include improved student knowledge and self-efficacy for integrating evidence-based lifestyle medicine guidelines into both their future professional practice and personal lives, with an emphasis on nutrition. In addition to these outcomes, this project aims to improve eating behaviors of student participants since healthy behavior role modeling may also positively contribute to patient receptivity of lifestyle counseling from their providers. Here, we describe the components of the lifestyle medicine curriculum and its impacts on student knowledge and self-efficacy in lifestyle medicine counseling, as well as personal eating behaviors.

## Methods

### Study Setting

The OU-TU School of Community Medicine Physician Assistant Program is jointly sponsored by the University of Oklahoma (OU) School of Community Medicine (SCM) and the University of Tulsa (TU) and is located in Tulsa, OK. The OU-TU SCM PA program is designed to prepare PA students with the knowledge and skills necessary to serve as well-rounded specialty or primary care providers, while learning a special skill set to improve the health of entire communities. The program operates in a state that ranks 44th poorest in premature death, 48th in cardiovascular disease death, 42nd in obesity, and 41st in diabetes prevalence—conditions that are all rooted in lifestyle-related health behaviors [13].

Students completing the Lifestyle Medicine course were entering their second year (13th month) of a 30-month PA curriculum. This transitional phase of the PA program marks the last semester before students begin clinical rotations. Completed coursework providing a foundational base for the course consisted of both basic science and clinical medicine courses, including Human Behavior, Public Health, Applied Physiology, Pharmacotherapeutics, Immunology, and a broad range of Clinical Medicine systems didactic coursework, including Gastroenterology.

### Program Development

The Lifestyle Medicine course was developed by the faculty of the OU College of Public Health (MSW) and the OUSCM Department of Emergency Medicine (LJW), in consultation with the Program Director of the OUSCM Physician Assistant Program (SDI). The 4-week, 2-credit hour course was developed to reinforce the four core lifestyle medicine competencies for primary care providers with an emphasis on nutrition instruction and other supporting lifestyle behaviors, such as physical activity and alcohol use. Materials developed by the American College of Lifestyle Medicine were also reviewed, including a webinar on implementing lifestyle medicine in an academic setting [14] and syllabus guide for developing lifestyle medicine coursework [15], as well as a list of recommended student readings on various lifestyle medicine and nutrition topics compiled by the ACLM [16] and the Tulane Goldring Center for Culinary Medicine (GCCM) [17]. The course activities were additionally designed to demonstrate adherence to several Accreditation Standards for PA Education, including B2.05, B2.06, B2.08, and B2.09 [3].

#### Didactic Components

Weekly 4-hour didactic lectures were developed by a registered dietitian (MSW) and taught by either the registered dietitian (MSW) or a MD physician (LJW). A medical student served as a graduate teaching assistant for the course (GCD) who co-facilitated weekly small group case study discussions.

Weekly assignments included small group patient case studies, quizzes, and weekly written reflections that were posted by students on the online course website. Students additionally reviewed pre-recorded nutrition lectures and self-study guides from the online “Health meets Food” courseware in preparation for that week’s cooking modules. The class concluded with a final exam and a small group project involving the development of a patient education material on a lifestyle medicine topic for use in clinical rotations (Table 1).

**Table 1:** Components of lifestyle medicine curriculum for physician assistant students

Week	Didactic lecture^1^	Culinary medicine topic^2^	Lifestyle medicine knowledge areas^1^	Assignments
Week 1	Assessment skills	• Introduction to culinary medicine • Sodium, potassium, and hypertension	• Evidence base for lifestyle medicine • 8 vital signs and related assessment tools	• Group case study • Written reflection • Quiz
Week 2	Management skills	• Weight management • Pediatrics	• Motivational interviewing • Weight management	• Group case study • Written reflection • Quiz
Week 3	Leadership role modeling	• Fats • Carbohydrates	• Cardiometabolic disease prevention and management • Diet and inflammation • Glycemic load	• Group case study • Written reflection • Quiz
Week 4	Use of office and community support	• Protein and vegetarian diets • Food allergies and intolerance	• Vegan and vegetarian nutrition • Decision support tools • Interprofessional teams	• Group case study • Written reflection • Final exam • Group patient handout

^1^Based on the American College of Lifestyle Medicine competencies for primary care physicians

^2^Modules from the “Health meets Food” curriculum that included full completion of the online courseware (videos, pre-readings) for each module and preparation of select recipes from those modules for each weekly cooking class

#### Culinary Medicine “Health meets Food” Curriculum

Consistent with the “Health meets Food” model, a formally trained chef (VC) co-facilitated the weekly cooking classes, with either the RD or MD serving as the healthcare provider co-instructor. Each culinary class was comprised of the following: 15 min of introduction of 8–10 recipes, instruction on applicable knife skills, cooking methods and techniques, and equipment usage; 75 min of instructor-supervised, student-led execution of recipes in small groups (2–3 recipes/group); and 30 min of tasting and discussion of how recipes and ingredients relate to the prevention and management of chronic conditions. Two medical student teaching assistants (GCD and EW) and two culinary art students from a local vocational training college additionally assisted with cooking class setup, demonstrations, and cleanup.

Due to time constraints, each weekly cooking class combined a selection of recipes from two separate “Health meets Food” modules (Table 1). The first class covered the “Health meets Food” modules titled, Introduction to Culinary Medicine and Sodium, Potassium, Hypertension. The second class covered the “Health meets Food” modules titled, Weight Management & Portion Control and Pediatric Diets. The third class covered the Fats and Carbohydrates modules. The fourth class covered the Food Allergy & Intolerance module and the Protein, Amino Acids, Vegetarian Diets, and Eating Disorders module. Prior to the first cooking class, all students were required to watch a “Health meets Food” courseware video on kitchen safety and sanitation. All cooking classes were delivered in a teaching kitchen designed for community use at the Tandy Family YMCA, which is located less than 2 miles away from the academic campus. Cooking classes were delivered weekly, back-to-back in 2-hour time blocks, with each class including a group of 12 PA students.

### Program Implementation and Associated Food Costs

The program was implemented in June 2018 with a class of second-year PA students (*n* = 24). Estimated total direct food cost to implement the full cooking class series, including 24 students and up to 7 staff members/student interns/assistants, was approximately $680, which is $4.47 per person per class.

### Program Evaluation and Measures

Prior to the first class session, second-year students enrolled at the OUSCM Tulsa PA Program (*n* = 24) or the University of Oklahoma Health Sciences Center (OUHSC) Oklahoma City PA Program (*n* = 48) were recruited via email with an invitation to complete an anonymous online survey. Both groups received an initial invitation email on May 23, 2018, with repeated reminders given to the intervention and control groups prior to the start of the first class on June 5, 2018. Upon conclusion of the 4-week course, students were contacted again with an invitation to complete a follow-up survey. The initial follow-up invitation email was sent on June 29, 2018, with repeated reminders to both intervention and comparison groups over 9 days. In each survey, students completed a self-generated anonymous ID [18] that was based on a combination of responses based on the model year of the respondent’s first car, number of older brothers, number of older sisters, birth year, and first initial of their maternal grandmother. Respondents received a $10 gift card for completing each survey. This study was reviewed and approved as exempt research by the University of Oklahoma Health Sciences Center Institutional Review Board. Informed consent was obtained from all individual participants included in the study.

Anonymous study data were collected using Qualtrics, while participant contact information for incentive payments were collected using REDCap electronic data capture tools hosted at the University of Oklahoma Health Sciences Center [19]. All anonymous study data were exported and analyzed using SPSS, version 24.

#### Demographics, Previous Nutrition Education, and Intended Specialty

Baseline demographic questions included age, gender, race, and ethnicity. Previous nutrition education was assessed, including completion of a major or minor degree in nutrition, as well as completion of any previous nutrition coursework, including and not including nutrition-related degrees. Respondents were also asked to report their intended specialty.

#### Current Health Behaviors

Respondents’ self-reported height and weight were used to calculate body mass index. We used items from the validated Fruit and Vegetable Food Behavior Checklist [20] and adapted the 14-item Mediterranean Diet Assessment Tool [21] for self-administration to assess adherence to a healthier eating pattern. The Fruit and Vegetable Food Behavior Checklist [20] assesses consumption patterns related to fruits and vegetables, including consumption of these foods as snacks, at main meals, servings per day, and consumption of citrus fruit or juice. The 14-item Mediterranean Diet Assessment Tool [21] assesses intake of food groups consistent with the Mediterranean diet, with points awarded for higher consumption of fruits, vegetables, nuts, beans, olive oil, fish, tomato-based sauces, wine; lower intake of red meats, animal fats, sugar-sweetened beverages, commercial sweets or pastries; and preference of white meat over red meat and olive oil over other culinary fats. We additionally asked whether students followed any specific dietary practices.

#### Lifestyle Medicine Counseling Knowledge and Confidence

We assessed knowledge and confidence to apply the modified 5A’s framework of lifestyle counseling (assess, advise, agree, assist, and arrange) [22] for the health behaviors of nutrition, exercise/physical activity, weight, smoking cessation, and alcohol use. Each step of the 5A’s framework was defined for students as follows: “Assess” was defined as “to assess a patient’s beliefs, behavior, and knowledge”; “Advise” was defined as “to advise a patient by providing specific information on health risks and benefits of change”; “Agree” was defined as “to collaboratively set goals based on a patient’s interest and confidence in their ability to change the behavior”; “Assist” was defined as “to assist a patient with identifying personal barriers, strategies, problem-solving techniques, and social/environmental support for behavior change”; and “Arrange” was defined as “to specify and arrange a plan for follow-up to support behavior change” [22]. For each of the five health behaviors, respondents separately rated their knowledge and confidence using Likert scale options ranging from not at all (1), slightly (2), somewhat (3), moderately (4), and extremely (5).

#### Qualitative Survey Components

In the follow-up survey, respondents who identified as Tulsa-based (intervention group) students were asked a series of open-ended questions to evaluate the culinary medicine component of the course, including, how have you applied culinary medicine techniques or food ingredients? Which recipes have you prepared? How have the culinary medicine classes impacted you personally? How have the culinary medicine classes shaped your future practice as a physician assistant? Students were also asked to share any recommendations or critiques for lifestyle medicine curriculum topics or opportunities, including nutrition, physical activity, tobacco cessation, alcohol counseling, stress management, sleep, or weight loss.

### Data Analysis

Self-generated identification codes were matched first on exact matches, then using an off-one system to identify additional matches. Codes that were not exact or off-one were not matched. This process is in line with commonly accepted methods for matching participants using self-generated identification codes.

#### Quantitative Survey Components

Race was classified as either non-Hispanic white or non-white. Body mass index (BMI) was calculated using self-reported height and weight. Mediterranean diet adherence was measured on a 0–14 scale with 1 point assigned to each item that met the adherence criteria for intake of that food [21]. If adherence criterion was not met for an item, a score of 0 was given. The 14 items were then summed for a total scale score. Each item comprising the 7-item Fruit and Vegetable Food Behavior Checklist was assigned between 1 and 4 points for a maximum total of 28 points, with higher scores indicating healthier fruit- and vegetable-related eating patterns [20].

We calculated frequencies for categorical variables and means and standard deviations for continuous variables. We first compared demographics, previous nutrition education, intended specialty, and body mass index of the intervention and comparison groups at baseline. We also compared outcomes of interest, including knowledge and confidence across all counseling topics (nutrition, physical activity, weight, smoking cessation, and alcohol) for each step of the 5A’s (assess, advise, agree, assist, and arrange), as well as Mediterranean diet adherence index and total fruit and vegetable behavior score, among intervention and comparison groups at baseline. We used independent samples *t*-tests to compare means between groups for continuous variables and chi-square tests for categorical variables.

To evaluate the impact of the lifestyle medicine course on student knowledge and confidence in the 5A’s of lifestyle counseling for each of the health behaviors assessed, we calculated mean differences in pre-post scores. We then conducted independent samples *t*-tests to compare the mean differences in scores for the intervention and control groups. Similarly, we calculated mean differences in pre-post scores for each of the two dietary assessment scales and then used independent samples *t*-tests to compare the mean differences in scores for the intervention and control groups. For all comparison statistics, the researchers used alpha of 0.05 to determine statistical significance.

#### Qualitative Survey Components

Qualitative survey data were independently reviewed by two members of the research team (MSW and EW). All responses were read thoroughly to ensure an understanding of the individual responses. Individual responses were then grouped according to similarity of responses by each reviewer. Themes were developed based on salient ideas that arose from the codes, and these identified themes were finalized through mutual consensus. The purpose of these analyses was to identify successful elements of the program according to participant perspectives, as well as to inform future improvements to the culinary medicine curriculum.

## Results

In total, 95.8% (23) of eligible intervention group (Tulsa) students and 50% (24) of the comparison group (Oklahoma City) students completed a baseline survey. In total, 91.7% (22) of eligible intervention group (Tulsa) students and 39.6% (19) of the comparison group (Oklahoma City) students completed a follow-up survey. Among respondents completing surveys, 11 respondents completed only a baseline survey and 5 respondents completed only a follow-up survey. Due to inconsistencies in self-generated anonymous IDs, 16 surveys could not be linked and were not included in the final pre-post analyses. One survey was deleted as it was deemed a duplicate response. A final sample size of 36 matched pairs were linked to allow for pre-post comparisons.

### Respondent Characteristics

We did not identify any significant demographic differences between intervention and comparison group respondents, with the exception of a younger mean age in the intervention group (*M* = 26.2, SD = 5.6) compared to the comparison group (*M* = 29.8, SD = 6.1), *p* = 0.041 (Table 2). Most students identified as non-Hispanic white (74.5%) and female (63.5%). While many students (38.3%) reported some previous exposure to nutrition through college coursework, more students (61.7%) reported no formal or informal nutrition education prior to the class.

**Table 2:** Baseline demographics of physician assistant students

Characteristic	Total (*n* = 47) *n* (%)	Intervention (*n* = 23) *n* (%)	Comparison (*n* = 24) *n* (%)	*p* value
Gender				
Male	14 (29.8)	6 (26.1)	8 (33.3)	0.587
Female	33 (63.5)	17 (73.9)	16 (66.7)	
Race/ethnicity				
White, non-Hispanic	35 (74.5)	18 (78.3)	17 (70.8)	0.559
Non-White^a^	12 (25.5)	5 (21.7)	7 (29.2)	
Year of school				
2nd year	47 (100)			
Intended specialty				
Primary care^b^	12 (25.5)	7 (30.4)	5 (20.8)	0.749
Medical specialty^c^	15 (31.9)	7 (30.4)	8 (33.3)	
Undecided	20 (42.6)	9 (39.1)	11 (45.8)	
Age				
Mean age (SD), range 22–47 years	28.02 (6.11)	*26.2 (5.6)*	*29.8 (6.1)*	*0.041*

^a^Non-White includes individuals identifying as black, Native American, Asian, and/or Hispanic

^b^Includes family medicine, internal medicine, obstetrics and gynecology, and pediatrics

^c^Includes anesthesiology, dermatology, emergency medicine, general surgery, neurology, otolaryngology, pathology, psychiatry, and sports medicine

Italics denote significant *p* value (< 0.05)

No significant differences were identified between intervention and comparison groups at baseline in knowledge and confidence level for executing each step of the 5A’s for nutrition counseling, with the exception of lower confidence in the “assistance” step among the intervention group (Table 3). No other significant differences were identified at baseline between intervention and comparison groups in knowledge and confidence level for executing each step of the 5A’s for the remaining four lifestyle behaviors assessed (results not shown).

**Table 3:** Baseline nutrition-related variables among physician assistant students

Characteristic	Total (*n* = 47) *n* (%)	Intervention (*n* = 23) *n* (%)	Comparison (*n* = 24) *n* (%)	*p* value
Nutrition education prior to PA school				
College major or minor in nutrition	7 (14.9)	4 (17.4)	3 (12.5)	0.770
Nutrition classes, no degree	11 (23.4)	6 (26.1)	5 (20.8)	
None	29 (61.7)	13 (56.5)	16 (66.7)	
	Mean (SD)	Mean (SD)	Mean (SD)	*P* value
Body mass index	24.67 (5.08)	23.8 (5.5)	25.5 (4.6)	0.249
5A’s nutrition knowledge				
Assess	3.23 (1.12)	3.14 (1.17)	3.32 (1.09)	0.595
Advise	3.36 (0.92)	3.32 (1.00)	3.41 (0.85)	0.747
Agree	3.36 (0.97)	3.27 (1.03)	3.45 (0.91)	0.539
Assist	3.25 (1.01)	3.09 (1.15)	3.41 (0.85)	0.304
Arrange	3.07 (1.09)	2.86 (1.17)	3.27 (0.99)	0.216
5A’s Nutrition Confidence				
Assess	3.23 (1.05)	3.05 (1.00)	3.41 (1.10)	0.257
Advise	3.02 (1.00)	2.82 (1.10)	3.23 (0.87)	0.178
Agree	3.23 (1.05)	3.09 (1.15)	3.36 (0.95)	0.397
Assist	3.25 (1.01)	*2.91 (0.97)*	*3.59 (0.96)*	*0.024*
Arrange	3.16 (1.08)	2.86 (1.13)	3.45 (0.96)	0.068
Nutrition behaviors				
Fruit and vegetable behavior score, sample range 11–27	19.62 (3.76)	19.74 (4.36)	19.50 (3.10)	0.834
Mediterranean index score, sample range 1–9	5.19 (1.88)	5.39 (1.97)	5.00, 1.82	0.483
Follows a special diet, *n* (%) Yes	7 (14.9)	5 (20.8)	2 (8.7)	0.243

Knowledge and confidence assessed using a 5-point Likert scale ranging from not at all (1), slightly (2), somewhat (3), moderately (4), to extremely (5)

Italics denote significant *p* value (< 0.05)

### Effectiveness

Compared to students in the comparison group, students in the intervention group improved more in knowledge and confidence in all of the 5A’s for nutrition counseling, *p* = 0.044 to < 0.001 (Table 4). Similarly, knowledge and confidence significantly increased in nearly all components of the 5A’s for physical activity and weight management counseling, except for no significant differences between groups in “assessment” confidence for physical activity and weight management and “advisement” knowledge for weight management (Table 4). Although not the primary focus of the course, knowledge and confidence gains in smoking cessation and alcohol counseling were also statistically higher in the intervention group with minimal improvement in the comparison group for most components of the 5A’s (Table 4). Although the differences between groups were not statistically significant, the fruit and vegetable index scores increased more among intervention students than comparison students, who experienced a decrease in the mean score for the Checklist. Both groups experienced slight increases in Mediterranean Diet Adherence score, but differences between groups were also not statistically significant (Table 5).

**Table 4:** Lifestyle medicine counseling knowledge and confidence, pre-post change scores between intervention (*n* = 21) and control (*n* = 14) physician assistant students

	Nutrition *M*△ (SD)			Physical activity *M*△ (SD)			Weight *M*△ (SD)			Smoking cessation *M*△ (SD)			Alcohol *M*△ (SD)		
	Intervention	Comparison	*p* value	Intervention	Comparison	*p* value	Intervention	Comparison	*p* value	Intervention	Comparison	*p* value	Intervention	Comparison	*p* value
Assess															
Knowledge	*1.29 (1.23)*	*0.71 (0.83)*	*0.003*	*0.71 (0.90)*	*0.07 (0.83)*	*0.041*	*0.71 (0.96)*	*0.07 (0.83)*	*0.048*	0.62 (0.92)	0.36 (1.34)	0.496	0.62 (0.86)	0.14 (1.17)	0.175
Confidence	*1.10 (1.30)*	*0.21 (0.97)*	*0.038*	0.95 (1.16)	0.29 (0.83)	0.073	0.85 (1.15)	0.36 (0.74)	0.161	0.76 (0.94)	0.36 (1.02)	0.263	0.86 (1.01)	0.43 (0.94)	0.216
Advise															
Knowledge	*0.90 (1.04)*	*0.21 (0.80)*	*0.044*	*0.81 (1.03)*	*0.07 (1.00)*	*0.043*	0.67 (0.80)	0.21 (0.80)	0.110	*0.71 (0.64)*	*0.00 (1.11)*	*0.022*	*0.90 (0.70)*	*0.00 (0.96)*	*0.003*
Confidence	*1.38 (1.12)*	*0.29 (0.73)*	*0.003*	*1.29 (1.23)*	*0.21 (0.70)*	*0.003*	*1.19 (1.25)*	*0.21 (0.70)*	*0.012*	0.71 (0.85)	0.29 (0.91)	0.164	*0.95 (0.86)*	*0.29 (0.61)*	*0.018*
Agree															
Knowledge	*1.05 (1.02)*	*0.21 (0.80)*	*0.015*	*0.95 (1.07)*	*0.14 (0.77)*	*0.021*	*0.90 (0.94)*	*0.21 (0.70)*	*0.026*	*0.76 (0.62)*	*0.14 (1.03)*	*0.033*	*0.95 (0.74)*	*0.21 (0.70)*	*0.006*
Confidence	*1.24 (1.09)*	*0.29 (0.73)*	*0.007*	*1.04 (1.02)*	*0.14 (0.77)*	*0.008*	*1.00 (1.10)*	*0.21 (0.70)*	*0.024*	1.00 (1.00)	0.36 (0.93)	0.064	*1.10 (0.94)*	*0.29 (0.83)*	*0.014*
Assist															
Knowledge	*1.29 (1.23)*	*0.07 (0.62)*	*0.001*	*1.14 (1.19)*	*0.14 (0.66)*	*0.008*	*1.10 (1.22)*	*0.21 (0.42)*	*0.014*	*0.90 (1.09)*	*0.07 (0.92)*	*0.025*	*1.19 (1.25)*	*0.07 (0.62)*	*0.001*
Confidence	*1.38 (1.02)*	*0.07 (0.83)*	*< 0.001*	*1.10 (1.10)*	*0.00 (0.68)*	*0.002*	*1.05 (1.20)*	*0.00 (0.68)*	*0.006*	*1.14 (1.06)*	*0.21 (0.80)*	*0.009*	*1.14 (1.11)*	*0.14 (0.66)*	*0.005*
Arrange															
Knowledge	*1.57 (1.29)*	*0.00 (0.88)*	*< 0.001*	*1.33 (1.32)*	*0.14 (0.77)*	*0.005*	*1.14 (1.28)*	*0.21 (0.80)*	*0.021*	*1.19 (1.08)*	*0.21 (0.89)*	*0.008*	*1.29 (1.15)*	*0.14 (0.77)*	*0.003*
Confidence	*1.38 (1.16)*	*0.14 (0.77)*	*< 0.001*	*1.00 (1.12)*	*0.00 (0.78)*	*0.007*	*1.10 (1.26)*	*0.07 (0.73)*	*0.010*	*1.19 (1.12)*	*0.29 (0.83)*	*0.015*	*1.24 (1.04)*	*0.21 (0.70)*	*0.003*

Knowledge and confidence assessed using a 5-point Likert scale ranging from not at all (1), slightly (2), somewhat (3), moderately (4), to extremely (5)

**Table 5:** Nutrition-related behaviors, pre-post change scores between intervention (*n* = 21) and comparison (*n* = 14) physician assistant students

	Intervention	Comparison	*p* value
Fruit and vegetable behavior score, *M* (SD)	0.91 (2.82)	− 0.64 (2.79)	0.121
Mediterranean index score, *M* (SD)	0.57 (1.63)	0.29 (1.14)	0.574

### Student Participant Qualitative Feedback

In response to the survey question about the application of culinary medicine techniques or ingredients, 19 students reported gaining at least one skill, with adding more vegetables to meals (*n* = 5), cutting an onion (*n* = 4), using less oil (*n* = 3), and improved cutting techniques (*n* = 3) being the most frequently reported. In response to the survey question about use of any recipes provided through the curriculum, nine students reported making at least one of the recipes at home. In response to the question about how the classes might shape his or her future practice as a PA, roughly half of all respondents provided a response to the question about how the class impacted them personally (*n* = 12). Overall, feedback was positive and included comments such as, “It made me more aware of what I was eating and how I could still eat yummy food that was nutritious,” and “It has helped encourage me to try more spices and vegetables that I used to be afraid of using.” Similarly, roughly half of all respondents provided a response about how the class will influence their future practice as a PA (*n* = 11). Illustrative quotes include, “It increases our ability to engage patients with empathy and understanding, and approach them from a place of shared experience,” “I now feel more confident about helping people identify barriers to changing their health behaviors and I feel like I can accurately give patients healthy recipes and strategies to have an overall better lifestyle,” and “I will 100% use my skills and knowledge and culinary medicine to educate my future patients.” Constructive feedback for the overall class was provided by ten students. Major areas for future improvements included adding more information on tobacco and alcohol counseling and condensing reading resources recommended by the “Health meets Food” online courseware.

## Discussion

This paper responds to a recent call to action for healthcare professions to advance basic and applied nutrition knowledge and skill sets that are specific to their discipline [23]. Specifically, we describe the development, implementation, and evaluation of nutrition-focused lifestyle medicine curriculum for PA students as one school’s response to this call. In the absence of explicit nutrition competencies for accredited PA programs, we developed new didactic content that aligned with four core competencies for lifestyle medicine in primary care and involved instruction on nutrition, physical activity, weight loss, stress management, mindfulness, sleep hygiene, tobacco, and alcohol guidelines. We further reinforced didactic nutrition content with a culinary medicine curriculum that was previously designed for medical students. To our knowledge, this is the first implementation of the “Health meets Food” curriculum in an accredited US PA program.

Overall, we found our curriculum to be effective in improving self-reported knowledge and confidence in patient counseling in the five lifestyle behaviors that we assessed; however, we noted the strongest gains were in nutrition counseling. This finding suggests that a hybrid approach involving didactic instruction combined with experiential processes may be required to maximize student mastery of lifestyle medicine. This pilot curriculum is consistent with guiding principles for healthcare professional training in nutrition, specifically (1) evidence-based nutrition guidelines for disease prevention and management, (2) the recognition and promotion of the role of the RD on the healthcare team, (3) nutrition education experiences that involve collaborative efforts with multiple stakeholders and innovative approaches for effective teaching, and (4) an introduction to evidence-based nutrition public health research that can be applied to patient care [1]. However, our approach does not meet the guiding principle for a longitudinal, integrated approach to nutrition education [1]. Although ideal, this guiding principle may not be practical for many PA programs for a number of reasons, such as a lack of equal buy-in across faculty and program administration. The impracticality of incorporating a full spectrum of nutrition competencies into existing medical education curricula led a recent study group to recommend one overarching goal for medical nutrition education, specifically that “All graduating medical students will assess nutritional status and manage the clinical encounter to facilitate a personalized approach for optimal health” [24]. This course was strategically placed in the semester immediately preceding clinical rotations, and further studies are needed to determine whether this curriculum improves student lifestyle medicine counseling behaviors.

A core competency of lifestyle medicine primary care providers is being personal champions of health through modeling of their own personal health behaviors. We were unable to identify significant gains in nutrition behaviors as a result of this intervention, although qualitatively, many students reported the curriculum’s impact on their own nutrition and likelihood of counseling in the future. Studies show that lifestyle behavior counseling is inconsistently performed in healthcare settings, as 72% of current tobacco users are not counseled on tobacco cessation by healthcare providers [25], and 64% of obese individuals do not receive weight management counseling [26]. Integrative and inventive programs, such as this hybrid PA curriculum, should be considered in all healthcare provider didactic training to improve rates of counseling and confidence in evidence-based recommendations by arming students with knowledge and personal experience. By educating future healthcare providers in the fundamentals of lifestyle medicine, we anticipate, based on our results, that these providers will incorporate lifestyle medicine in their practice and begin to build a healthy lifestyle-focused culture in medicine. Preventive medicine through lifestyle change is an example of value-based care due its low cost of implementation yet strong potential for high impact, and leaders in the field encourage integration of lifestyle medicine in routine medical practice [4, 27].

This study includes several limitations. First, we were unable to conduct a second follow-up survey to assess retention of knowledge and confidence over time. Additionally, our evaluation did not include an objective assessment of nutrition counseling skills. Third, the condensed course was delivered over a short period of time, which may have limited the potential of the curriculum to impact participants’ personal eating behaviors. Larger studies are needed to determine the intensity and mode of instruction needed to effect healthy nutrition behavior change among future healthcare providers, as well as to objectively evaluate the curriculum’s impact on patient counseling behaviors. Limitations of the study include the short length of the course, and a greater impact on personal eating behaviors might be seen in a longer intervention, possibly combined with extracurricular student wellness activities. Larger studies are needed to determine the intensity and mode of instruction needed to effect healthy nutrition behavior change among future healthcare providers.

## Curriculum Implications for PA Programs

A potential limitation to the feasibility of implementing a nutrition-focused lifestyle medicine course into a PA program is the inclusion of additional educational objectives into an existing intensive curriculum. Many administrators of PA programs are hesitant to expand program length, which may potentially further encroach on medical school duration, and thus may conflict with the initial intent for the development of the profession and the goal of efficiently training clinicians in a shorter timeframe to meet healthcare shortages. PA programs are often already challenged in adequately delivering content required to meet accreditation standards as well as for successful passage of national certification and for clinical competency in delivering healthcare in technologically advanced healthcare systems. In addition, funding for a nutrition-focused course, as implemented with hands-on cooking instruction from a formally trained chef assisted by a registered dietitian or licensed healthcare provider, is a limiting factor without specific designated funding. A less cost-intensive course limited to didactic instruction would diminish the benefits of the experiential learning, yet increasing the financial strain placed on students in relation to increased tuition and fees would not be ideal. Acceptability of the curriculum implementation from students and faculty involves education on the clinical significance of improved patient outcomes from nutritional counseling, while addressing a perceived detraction from traditional clinical medicine instruction. However, PA education has a history of responding to the needs of healthcare systems. The growing rates of chronic conditions faced by today’s healthcare providers require a professional ownership of a prevention-focused approach to patient care.

## Conclusion

This nutrition-focused lifestyle medicine course directly addressed several accreditation standards for PA programs, particularly those related to providing instruction in patient education skills and assisting patients to modify their behaviors to more healthful patterns. It also provided the OU-TU School of Community Medicine PA program with the opportunity to achieve its overarching goal of training providers to transformatively shape the health of entire communities. PA school is a formative time to both ensure that foundational competencies in primary care are adequately developed and to shape the practice philosophy of future healthcare providers regardless of future specialty practice. Lifestyle medicine curricula represent one innovative opportunity for impacting a PA provider’s ability to adopt healthy self-care behaviors as well as apply evidence-based strategies for delivering quality patient care. Longitudinal studies of students who receive lifestyle medicine training are needed to determine the impact of these curricula, specifically whether these efforts are useful in shaping health behaviors of providers, as well as their ability to proactively impact the health of the community through lifestyle behavior change.
